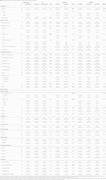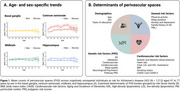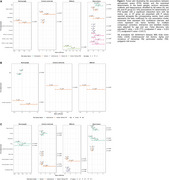# Analysis of perivascular spaces highlights regional enlargement by sex, amyloid status, AD genetic predisposition and specific risk factors

**DOI:** 10.1002/alz70856_102667

**Published:** 2025-12-25

**Authors:** Alba Fernández‐Bonet, Patricia Genius, Blanca Rodríguez‐Fernández, Armand González Escalante, Jordi Huguet, Manel Esteller, Marta Cirach, Mark Nieuwenhuijsen, Mariateresa Buongiorno, Gonzalo Sánchez‐Benavides, Oriol Grau‐Rivera, David Vállez‐Garcia, Arcadi Navarro, Tavia E Evans, Natalia Vilor‐Tejedor

**Affiliations:** ^1^ Barcelonaβeta Brain Research Center (BBRC), Pasqual Maragall Foundation, Barcelona, Spain; ^2^ Hospital del Mar Research Institute, Barcelona, Spain; ^3^ Centre for Genomic Regulation (CRG), Barcelona Institute of Science and Technology (BIST), Barcelona, Spain; ^4^ Hospital del Mar Research Institute (IMIM), Barcelona, Spain; ^5^ BarcelonaBeta Brain Research Center (BBRC), Barcelona, Spain; ^6^ Institució Catalana de Recerca i Estudis Avançats (ICREA), Barcelona, Spain; ^7^ Josep Carreras Leukaemia Research Institute (IJC), Badalona, Barcelona, Spain; ^8^ Physiological Sciences Department, School of Medicine and Health Sciences, University of Barcelona (UB), Barcelona, Catalonia, Spain; ^9^ Centro de Investigación Biomédica en Red de Epidemiología y Salud Pública (CIBERESP), Madrid, Spain; ^10^ ISGlobal, Barcelona Institute for Global Health ‐ Campus MAR, Barcelona Biomedical Research Park, Barcelona, Spain; ^11^ Neurovascular Diseases Research Group, Vall d'Hebron Research Institute, Barcelona, Spain; ^12^ Neurology Department, Vall d'Hebron University Hospital, Barcelona, Spain; ^13^ Centro de Investigación Biomédica en Red de Fragilidad y Envejecimiento Saludable (CIBERFES), Instituto de Salud Carlos III, Madrid, Spain; ^14^ Department of Radiology and Nuclear Medicine, Amsterdam UMC, Amsterdam, Netherlands; ^15^ Institute of Evolutionary Biology (CSIC‐UPF), Department of Experimental and Health Sciences, Universitat Pompeu Fabra, 08003, Barcelona, Spain; ^16^ Radboud University Medical Center, Nijmegen, Netherlands

## Abstract

**Background:**

Perivascular spaces (PVS), fluid‐filled compartments around brain blood vessels, are markers of small vessel disease and overall brain health. Key to the glymphatic system, they aid in waste clearance and may influence neuroinflammation. MRI‐defined PVS have been linked to stroke, cognitive decline, and Alzheimer's disease (AD). This study examined determinants of PVS burden in cognitively unimpaired individuals at AD risk.

**Method:**

We analyzed PVS burden through T2‐weighted MRI scans for 322 individuals from the ALFA+ study across four brain regions: basal ganglia (BG), centrum semiovale (CSO), midbrain (MB), and hippocampus (HP), using a validated machine learning‐based automatic rating protocol [Table 1]. Poisson and negative binomial regression models were used to examine the influence on regional PVS counts of demographics, cardiometabolic and mental health factors, polygenic risk scores associated with AD‐related pathways, metabolic traits, and sleep disturbances. Interaction and stratified models by sex, amyloid status, and CSF amyloid/tau biomarker classification framework (AT) groups were assessed to explore potential modifiers.

**Result:**

In the CSO, significant age‐related increases in PVS counts and consistent sex differences were observed, with men showing higher counts [Figure 1]. In the HP, amyloid models showed that sex, sleep duration, and *APOE*‐ε4 were associated with greater PVS enlargement among A+ individuals. Nominal trends included systolic blood pressure, and CAIDE‐II (HP); particulate matter (PM_2.5_) (BG, CSO), and genetic predisposition to AD through cholesterol efflux (MB), all showing greater enlargement in A+ individuals [Figure 2A]. Sex‐specific models revealed a significant inverse association between BMI and PVS burden in men (BG), and nominal effects of genetic predisposition through complex lipoprotein (BG) and amyloid (CSO, MB) also in men [Figure 2B]. AT‐related models highlighted associations for smoking in A+T+ individuals (CSO) and systolic blood pressure in A‐T‐ (HP), with additional nominal trends for PM_2.5_ (BG), sleep duration and depression (HP), and cholesterol efflux (MB) in A+T+ and A+T‐ [Figure 2C].

**Conclusion:**

These findings highlight sex‐ and disease stage‐specific influence of risk factors on PVS, as well as the regional variability of these associations, underscoring the absence of an uniform brain‐wide effect.